# The mechanism of vitamin D3 in preventing colorectal cancer through network pharmacology

**DOI:** 10.3389/fphar.2023.1192210

**Published:** 2023-05-17

**Authors:** Kang Rong, Qingmin He, Shu Chen, Yong Yu, Lu Mei, Yang Mi, Liufan Mu, Mingyang Zhu, Mengjiao Nan, Xiaoyang Zhang, Zhaoyang Wan, Huang Huang, Pengyuan Zheng

**Affiliations:** ^1^ Henan Key Laboratory of Helicobacter Pylori and Microbiota and Gastrointestinal Cancer, Marshall B. J. Medical Research Center of Zhengzhou University, The Fifth Affiliated Hospital of Zhengzhou University, Zhengzhou, China; ^2^ Department of Gastroenterology, The Fifth Affiliated Hospital of Zhengzhou University, Zhengzhou, China; ^3^ The First Clinical Medical School of Henan University of Chinese Medicine, Zhengzhou, China

**Keywords:** vitaminD3, CRC, chemoprevention, network pharmacology, molecular docking

## Abstract

**Objective:** Colorectal cancer (CRC) is a common cancer that cannot be detected at an early stage and is a major challenge in oncology research. Studies have shown that vitamin D3 has some anti-cancer and preventive effects on colorectal cancer, but the exact anti-cancer mechanism is not clear. We applied the relevant research methods of network pharmacology to speculate and validate the possible potential pharmacological mechanisms of vitamin D3 for the prevention of colorectal cancer, and to provide more theoretical support for the clinical anticancer effects of vitamin D3.

**Methods:** The relevant targets for vitamin D3 and CRC were obtained from the database of drug and disease targets, respectively. The target of vitamin D3 and the target of colorectal cancer were taken to intersect to obtain common targets. Then, the PPI network was constructed. In addition, the pathways of drug-disease interactions were predicted by GO and KEGG enrichment analysis. Finally, the obtained results were verified to ensure the reliability of the experiments.

**Results:** 51 targets of vitamin D3 for the prevention of colorectal cancer were obtained. The 10 core targets were obtained from the PPI network. The 10 core targets include: ALB, SRC, MMP9, PPARG, HSP90AA1, IGF1, EGFR, MAPK1, MAP2K1 and IGF1R. The core targets were further validated by molecular docking and animal experiments. The results suggest that vitamin D3 plays a key role in the prevention of CRC through core targets, PI3K-Akt pathway, HIF-1 pathway, and FoxO pathway.

**Conclusion:** This study will provide more theoretical support for vitamin D3 to reduce the incidence of CRC and is important to explore more pharmacological effects of vitamin D3.

## 1 Introduction

Colorectal cancer (CRC) is a malignant tumor that has the third highest incidence rate among cancers and the second highest mortality rate among malignant tumors (Sung et al., 2021). In China, CRC ranks fifth in the incidence of malignant tumors in men and fourth in the incidence of malignant tumors in women. CRC ranks fifth in both male malignancy mortality and female malignancy mortality (Chen et al., 2016). Despite extensive research, the pathogenesis of CRC remains unclear (Thanikachalam and Khan, 2019). With the improvement of treatment methods and medical devices, the risk of CRC has already decreased, but the disease burden of CRC is still heavy, and more prevention strategies are needed (Schoen et al., 2012; Nishihara et al., 2013; Shaukat et al., 2013; Fakih, 2015; Doubeni et al., 2018; Levin et al., 2018). The concept of chemoprevention was first introduced in 1976. Chemoprevention of cancer usually involves the use of synthetic drugs to reduce the incidence of cancer and even reverse pre-cancerous and carcinogenic processes (Sporn, 1976). Currently, drugs that can have a chemopreventive effect on CRC include statins, drugs that target metabolic pathways, aspirin,non-aspirin non-steroidal anti-inflammatory drugs, and vitamins and minerals (Katona and Weiss, 2020). Vitamin D maintains relatively stable levels of calcium in the body’s serum, and calcium salts deposited in certain areas contribute to the formation of bone. Several epidemiological studies have shown that patients with inadequate vitamin D intake have an increased risk of developing cancer (Holick, 2004; Bikle, 2016; Wang et al., 2017). In addition, studies have shown that vitamin D has a specific inhibitory effect on cancer (Harris and Go, 2004; Fleet et al., 2012; Feldman et al., 2014; Giammanco et al., 2015). The vitamin D receptor is expressed in a variety of cells in the body. By targeting the vitamin D receptor to initiate intracellular signaling regulatory mechanisms, it regulates complex intracellular signaling and ultimately inhibits CRC (Leyssens et al., 2013; Baron JA et al., 2015). Studies have shown that patients with low levels of vitamin D in the serum are more likely to develop CRC. Thus, vitamin D supplementation has a role in improving patient prognosis and preventing the development of CRC (Terry et al., 2002; Mccullough et al., 2003; Ma et al., 2011), although these results are based primarily on studies of the distal colon and rectum (Zheng et al., 1998; Wu et al., 2002). This study reviewed the literature related to vitamin D and CRC and ultimately concluded that vitamin D has an inhibitory effect on CRC. However, the pathway is still unclear and more research is needed.

## 2 Materials and methods

### 2.1 Overview of the present study

**FIGURE 1:**
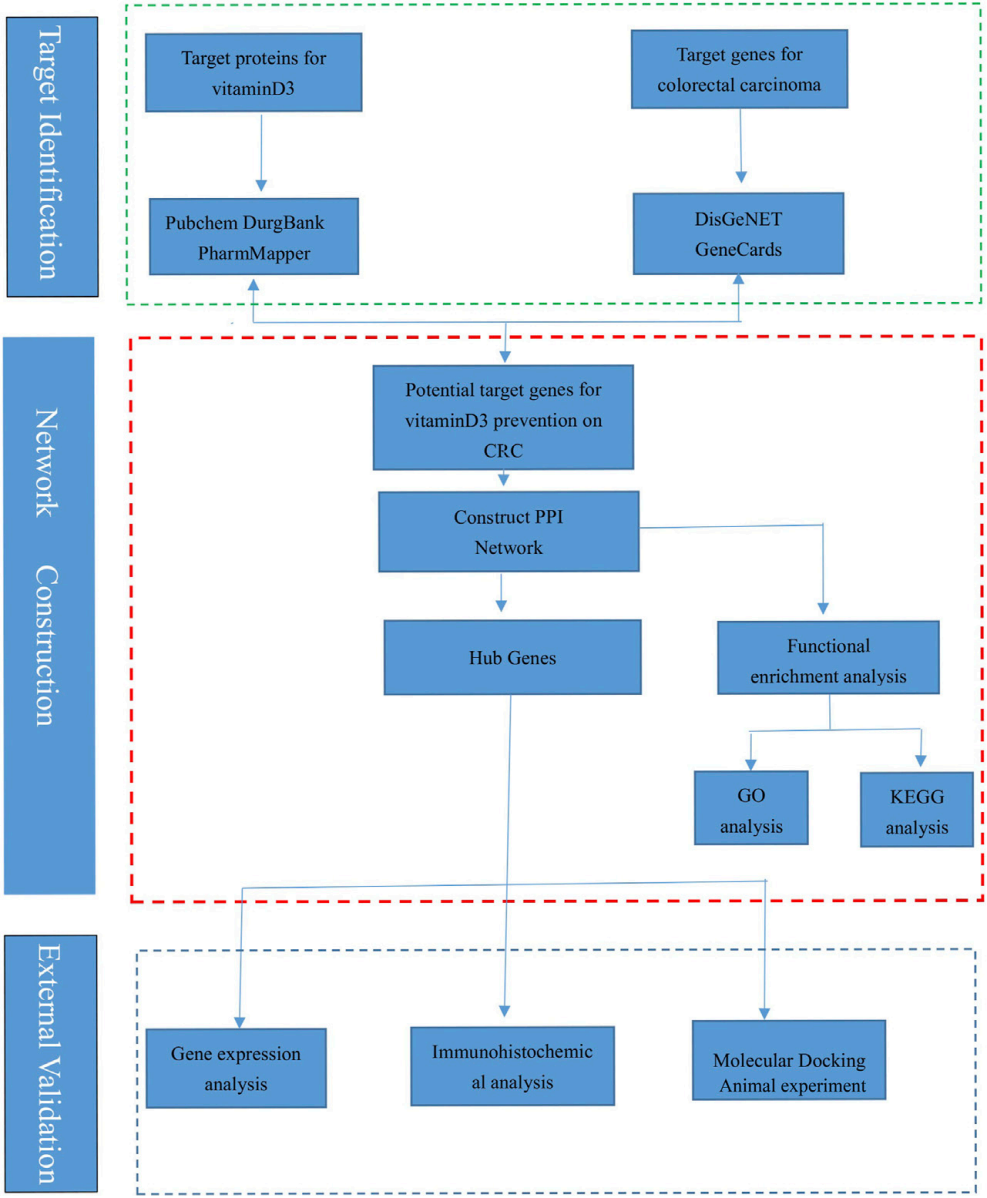
The flow chart of this research.

The research process is shown (Figure 1), and the databases involved are listed (Table 1).

**TABLE 1:** Website information of the database involved in the study of vitamin D3 prevention of colorectal cancer.

Name	URL
PubChem	https://pubchem.ncbi.nlm.nih.gov/
PharmMapper	http://lilab-ecust.cn/pharmmapper/
Drugbank	https://go.drugbank.com/
Targetnet	http://targetnet.scbdd.com/
DisGeNET	https://www.disgenet.org/
GeneCards	https://www.genecards.org/
GEPIA	http://gepia.cancer-pku.cn/
HPA	https://www.proteinatlas.org/
Uniprot	https://www.uniprot.org/
STRING	https://cn.string-db.org/
DAVID	https://david.ncifcrf.gov/
Venny 2.1.0	https://bioinfogp.cnb.csic.es/tools/venny/index.html
RCSB PDB	https://www.rcsb.org/
Bioinformatics	http://www.bioinformatics.com.cn/
Cytoscape	https://cytoscape. org/
cBioPortal	https://www.cbioportal.org/
Kaplan-Meier Plotter	https://kmplot.com/analysis/

### 2.2 Molecular properties of vitaminD3

Vitamin D3 is a class of steroid hormones that are usually synthesized by the body in response to UV radiation or supplemented by food. Studies have shown that vitamin D3 is necessary to maintain calcium homeostasis and bone mineralization in the body. Binding of vitamin D3 and its receptors can regulate specific gene expression and signal transduction in the body, exerting biological effects. The properties of vitamin D3 are shown (Table 2), and the data are from PubChem.

**TABLE 2:** Detailed molecular properties of vitamin D3 in PubChem.

Name	Details
PubChem CID	5280795
Molecular Formula	C_27_H_44_O
Synonyms	cholecalciferol
Molecular Weight	384.6
CAS	67–97–0
XLogP3-AA	7.9
Hydrogen Bond Donor Count	1
Hydrogen Bond Acceptor Count	1
Rotatable Bond Count	6
Topological Polar Surface Area	20.2 Å^2^
Heavy Atom Count	28
Complexity	610
Covalently-Bonded Unit Count	1

### 2.3 Target of vitamin D3 was obtained

The English name of the drug was input to get its details in PubChem (https://pubchem. ncbi.nlm.nih.gov/) (Kim et al., 2021). The SDF format file of the drug was obtained from PubChem and then imported into PharmMapper (http://lilab-ecust.cn/pharmmapper/) (Liu et al., 2010). In PharmMapper, the screening criteria are as follows: Generate Conformers:Yes, Maximum Generated Conformations:300, Number of Reserved Matched Targets (Max 1,000):300. The canonical smiles of the drug was searched to obtain the target of vitamin D3 in Targetnet (http://targetnet.scbdd.com/) (Yao et al., 2016), and the English name of the drug was searched to obtain the target of vitamin D3 in Drugbank (https://go.drugbank.com/) (Wishart et al., 2018). The targets of the drug were converted into gene names in UniProt (https://www.uniprot.org/) (UniProt Consortium, 2021). Finally, the gene names were merged and de-duplicated to obtain the potential targets of vitamin D3.

### 2.4 CRC-related targets were obtained

The targets were obtained in DisGeNET (https://www.disgenet.org/) (Pinero et al., 2020) and GeneCards (https://www.genecards.org/) (Stelzer et al., 2016). The targets were merged and de-duplicated to be the related targets of CRC.

### 2.5 Screening of intersection targets and construction of PPI network

The targets of vitamin D3 and CRC-related targets were taken intersected in Venny 2.1.0 (https://bioinfogp.cnb.csic.es/tools/venny/index.html). Finally, the intersecting targets obtained are potential targets of vitamin D3 for CRC prevention. The intersection targets are imported into String (https://cn.string-db.org/) to construct the PPI network. The tsv file of the PPI network was obtained and imported into Cytoscape 3.9.0 (https://cytoscape. org/; Version 3.9.0) (Doncheva et al., 2019) for analysis and visualization. In cytohubba, the core targets are obtained according to the importance of the nodes. The core targets were visualized.

### 2.6 GO and KEGG enrichment analysis

Core targets were imported into David (https://david.ncifcrf.gov/) (Huang et al., 2007) to obtain the results of GO and KEGG enrichment analysis (Ashburner et al., 2000; Kanehisa et al., 2021). The screening criteria were *p* < 0.05 and the enriched signaling pathway contained multiple core targets. The first 10 entries of BP, CC and MF were selected in this study, respectively. The collated data were imported into the bioinformatics (http://www.bioinformatics. com. cn/) for visualization and analysis.

### 2.7 Construction of drug-target-pathway network

*p* < 0.05 was used as a screening criterion, and the top 20 signaling pathways were selected based on the ranking of *p*-values from smallest to largest. Data of vitamin D3, signaling pathways and core targets were made into network. xlsx and type. xlsx and the files were imported into Cytoscape for visualization. The vitamin D3-core target-pathway network was constructed. The red triangle represents vitamin D3, the blue diamond represents the core target, and the green circle represents the signaling pathway.

### 2.8 Molecular docking

#### 2.8.1 The process of molecular docking

To explore the possible mechanisms of vitamin D3 action on CRC. The strength and pattern of interactions between vitamin D3 and core targets were assessed by molecular docking in this study. The PDB ID of the core target was obtained from the RCSB PDB and its 3D structure was downloaded in PDB format. Molecular docking was performed using Discovery studio (2019 version) and the “Prepare Ligands” module was used to obtain 3D structures in this study. After removing the water of crystallization, the “Prepare Protein” module is used to remove multiple conformations of the target protein by adding hydrogen atoms. Then, the fast molecular docking option is selected in the “LibDock” module. The software calculates the corresponding docking scores in the columns of the LibDockScore. The docking score as a judgment criterion was used to evaluate the affinity of the target protein and the drug.

#### 2.8.2 Reliability of molecular docking was verified

To verify the reliability of molecular docking, the original ligands of 10 target protein complexes were separated and then reattached to the pockets of the complexes in this study. The root-means-square deviation (RMSD) of the ligand conformation after ligand docking and the ligand conformation in the original crystal structure were calculated. The RMSD value less than 2.0Å indicates that the docking method can well reproduce the original ligand-receptor binding mode and the docking parameters are reasonably set.

### 2.9 External validation of core targets

#### 2.9.1 Expression at the gene level

In GEPIA (http://gepia.cancer-pku.cn), the expression information of the core targets were obtained. The relevant information of core targets for different populations and different stages of cancer were obtained in the “Expression DIY” module (Tang et al., 2017).

#### 2.9.2 Expression at the protein level

Differences of core targets were studied at the protein expression level. The relevant data from the Human Protein Atlas database (https://www.proteinatlas.org/) (Uhlen et al., 2015) were analyzed. The core targets were analyzed and compared using the relevant data from this database to make further conclusions.

#### 2.9.3 Alteration of genetic information in CRC

The 105 colon cancer (CPTAC - 2 prospective, cell 2019) samples in the database were analyzed using cBioPortal (https://www.cbioportal.org/) (Wu et al., 2019). We obtained the genetic information of the core targets (He et al., 2023). In addition, the mutation sites and mutation types of the core targets were obtained from this database.

#### 2.9.4 Presence of core target proteins in other cancers

In GEPIA, the “General” module was selected and the core targets were entered to obtain the distribution and expression levels of the core targets in different cancers. The obtained results were presented in the form of a dot plot.

#### 2.9.5 The effect of core targets on survival in CRC

To explore the impact of core targets on overall survival in CRC, Kaplan-Meier Plotter (https://kmplot.com/analysis/) was accessed. After the core targets were entered, the results of the overall survival of each target were obtained in this database.

#### 2.9.6 Animal experimental verification

##### 2.9.6.1 Experimental animals

Eighteen 7-week-old male C57BL/6N mice were procured from Beijing Weitong Lihua Experimental Animal Technology Co. The mice were randomly divided into control group, model group and vitamin D3 intervention group, 6 mice in each group. The experimental conditions included constant temperature, pressure and humidity, and 12 h of light per day. Mice were fed with Co60 irradiated sterilized maintenance feed. The animal experiments were approved by the Animal Ethics Committee of the Fifth Affiliated Hospital of Zhengzhou University (Ethics No: KY2023007).

##### 2.9.6.2 Reagents

Vitamin D3 (Item No.: B2065) was purchased from APExBIO(Houston,TX). Azoxymethane (AOM, item number: 0218397125) was purchased from Abe Medical Device Trading (Shanghai) Co., LTD., and Dextran sulfate sodium (DSS, item number MB5535) was purchased from Meilan Biotechnology (Dalian) Co., LTD. RNAiso Plus was purchased from Takara bio, Japan, and RIPA cell lysate and protease inhibitors were purchased from Dingguo Changsheng Biotechnology (Beijing) Co., LTD. Protein phosphatase inhibitors were purchased from bimake Biotechnology Co., LTD. HiScript®II 1st Strand cDNA Synthesis Kit and ChamQ universal SYBR qPCR Master Mix were purchased from Novizam Biotechnology (Nanjing) Co., LTD. The corresponding primers of the core target were synthesized by Sangon Biotech (Shanghai).

##### 2.9.6.3 Animal grouping and modeling methods

A completely randomized design was used to divide the mice into three groups. Physiological saline control group (Nacl): 6 mice, model group (AOM + DSS): 6 mice, vitamin D3 intervention group (AOM + DSS + vitaminD3): 6 mice. After 1 week of adaptive feeding, the mice were gavaged with vitamin D for 2 continuous weeks at a dose of 60 IU/g per week, 3 times per week. At week 4, mice of the model and vitamin D3 intervention groups were given a single intraperitoneal injection of AOM (10 mg/kg). At weeks 5, 8 and 11, the model and vitamin D3 intervention groups were given drinking water containing 2% DSS for 5 days and normal drinking water for the rest of the day. The mice were weighed twice a week. Mice were sacrificed at week 15, and colorectal tissues were collected for RT-qPCR experiments.

##### 2.9.6.4 RT-qPCR

Gene expression of the core targets were detected in colorectal tissue by RT-qPCR. Total RNA was extracted from mouse intestinal tissue following the tissue RNA extraction protocol. The purity of RNA was detected by NanoDrop ND-2000 spectrophotometer and the ratio of A260/A280 was recorded. The reverse transcription reactions were performed on a common PCR instrument using the HiScript®II 1st Strand cDNA Synthesis Kit. Real-time fluorescence quantitative PCR was performed on a Light Cycler 480II real-time fluorescence quantitative PCR instrument. The 2^−ΔΔCT^ method was used to calculate the expression of core targets. The corresponding primer sequences of the core targets were listed (Table 3).

**TABLE 3:** Primer sequences corresponding to core targets.

Gene name	Forward primer (5'→3′)	Reverse primer (5'→3′)
*GAPDH*	TGA​CCT​CAA​CTA​CAT​GGT​CTA​CA	CTT​CCC​ATT​CTC​GGC​CTT​G
*ALB*	TCC​AAA​CCT​CCG​TGA​AAA​CTA​TG	TGT​GTT​GCA​GGA​AAC​ATT​CGT
*SRC*	GAA​CCC​GAG​AGG​GAC​CTT​C	GAG​GCA​GTA​GGC​ACC​TTT​TGT
*MMP9*	GCA​GAG​GCA​TAC​TTG​TAC​CG	TGA​TGT​TAT​GAT​GGT​CCC​ACT​TG
*PPARG*	GGA​AGA​CCA​CTC​GCA​TTC​CTT	GTA​ATC​AGC​AAC​CAT​TGG​GTC​A
*HSP90AA1*	GAC​GCT​CTG​GAT​AAA​ATC​CGT​T	TGG​GAA​TGA​GAT​TGA​TGT​GCA​G
*IGF1*	CTG​GAC​CAG​AGA​CCC​TTT​GC	GGA​CGG​GGA​CTT​CTG​AGT​CTT
*EGFR*	ATG​AAA​ACA​CCT​ATG​CCT​TAG​CC	TAA​GTT​CCG​CAT​GGG​CAG​TTC
*MAPK1*	GGT​TGT​TCC​CAA​ATG​CTG​ACT	CAA​CTT​CAA​TCC​TCT​TGT​GAG​GG
*MAP2K1*	AAC​GGT​GGA​GTG​GTC​TTC​AAG	CGG​ATT​GCG​GGT​TTG​ATC​TC
*IGF1R*	CTT​CTA​CAA​CTA​CGC​ACT​GGT​C	TCG​GCG​TTC​TTC​TCA​ATC​CTG

## 3 Results

### 3.1 Target identification and analysis

**FIGURE 2:**
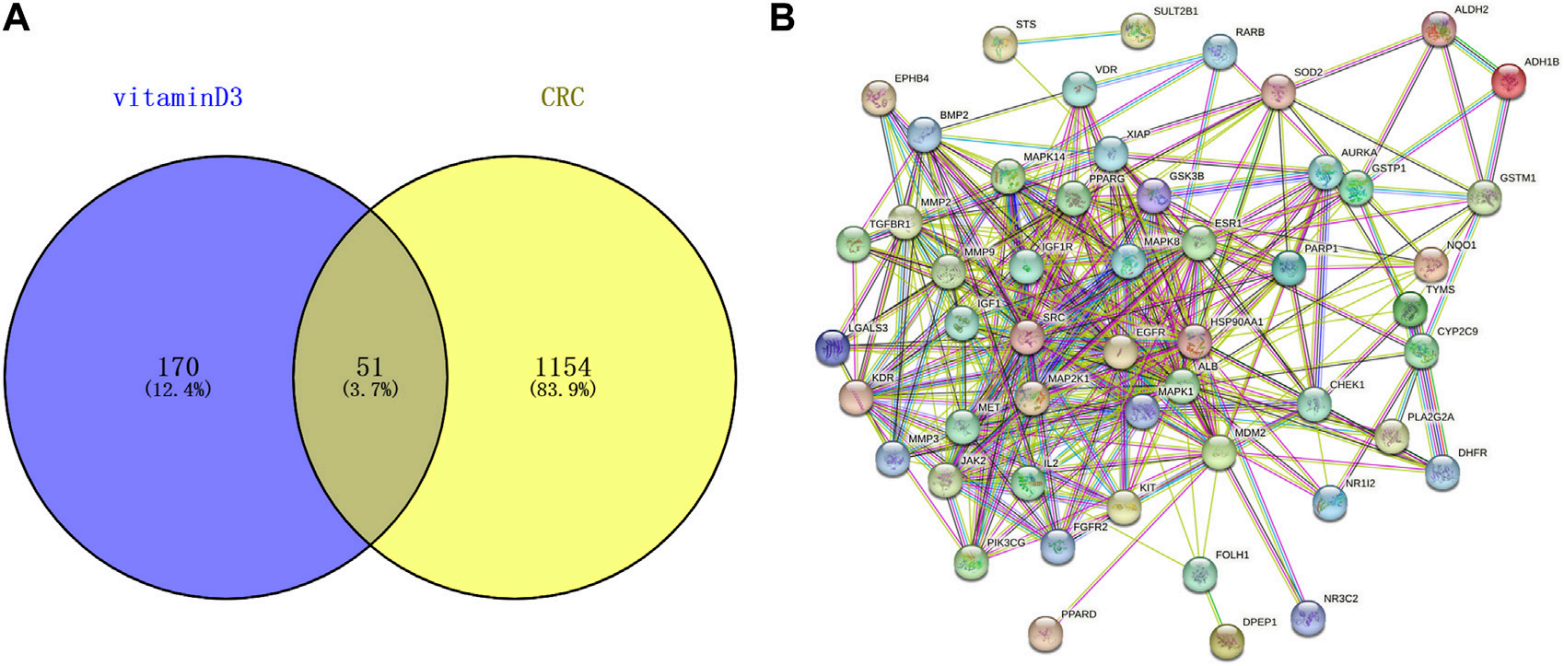
Information related to CRC chemoprevention. **(A)** Venn diagram of intersection targets between vitamin D3 and CRC **(B)** Establishment of PPI network for intersection targets.

221 targets of vitamin D3 were obtained in PharmMapper, DrugBank and Targetnet databases. 1205 CRC-related targets were obtained in the DisGeNET and GeneCards databases. The targets of vitamin D3 and CRC-related targets were imported into Venny 2.1.0, and 51 intersecting targets were obtained (Figure 2A).

### 3.2 Establishment of PPI network and selection of core targets

**FIGURE 3:**
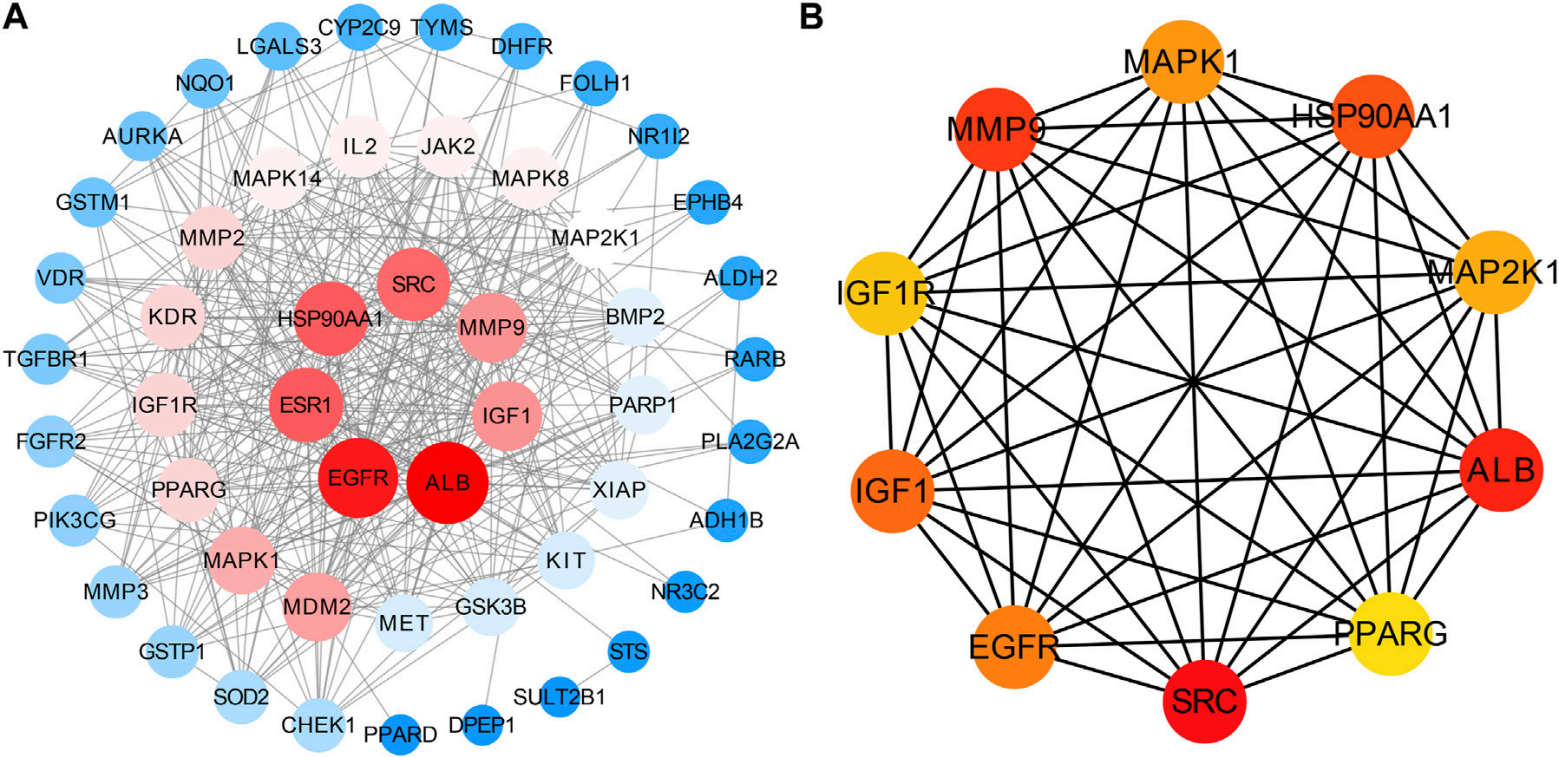
Visual results of intersection and core targets. **(A)** Graphical display of intersection targets **(B)** Visualization of core target genes.

51 intersecting targets were input to String, and a PPI network (Figure 2B) containing 51 nodes and 369 edges was established. The tsv file of the PPI network was downloaded and imported into Cytoscape 3.9.0 for visualization and analysis (Figure 3A). The intersecting targets were ranked from largest to smallest according to the degree value using the Cytohubba plugin. The top 10 intersecting targets were selected as core targets for vitamin D3 prevention of CRC. The core targets are visualized (Figure 3B). The circles represent the core targets for screening, and the redder the color of the circle, the greater the importance (Chin et al., 2014). The 10 core targets include ALB, SRC, MMP9, PPARG, HSP90AA1, IGF1, EGFR, MAPK1, MAP2K1 and IGF1R.

### 3.3 Results of enrichment analysis

**FIGURE 4:**
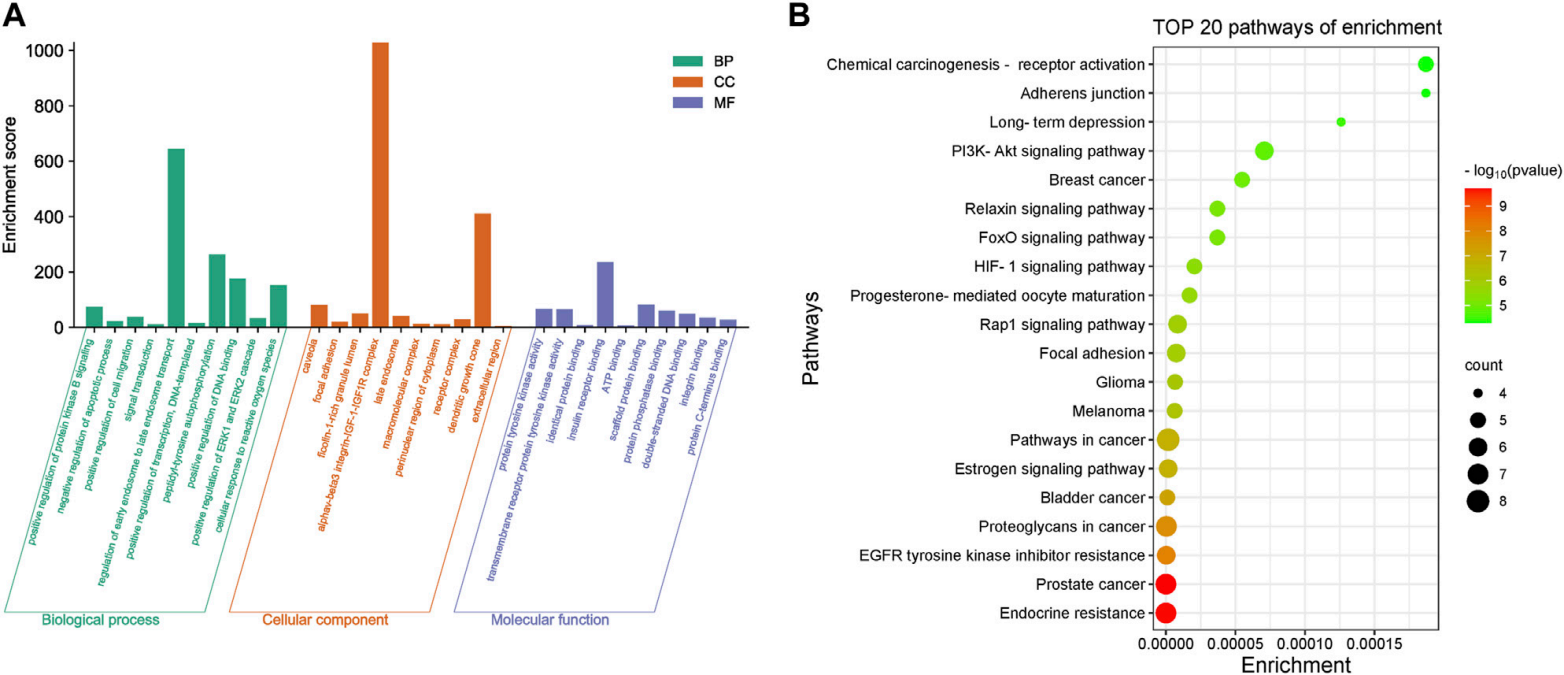
Enrichment results of core targets **(A)**. Visualization results of GO enrichment analysis. **(B)** Visualization results of KEGG enrichment analysis.

Core targets were imported into DAVID for GO and KEGG enrichment analysis. The results of GO enrichment analysis of core targets included 134 entries, BP(Biological Process):90, CC(Cellular Component):23, MF(Molecular Function):21. The top 10 items in each category were selected for visual analysis (Figure 4A). The biological processes involved in the 10 core targets were mainly endosome transport, peptidyl-tyrosine autophosphorylation, and positive regulation of DNA binding. They mainly act on the alphav-beta3 integrin-IGF-1-IGF1R complex, dendritic growth cone. They are involved in molecular functions including insulin receptor binding, scaffold protein binding, protein tyrosine kinase activity, etc. 80 signaling pathways were identified by KEGG enrichment analysis of core targets, and 20 important signaling pathways were visualized and analyzed (Figure 4B). The main signaling pathways involved include the HIF-1 signaling pathway, PI3K-Akt signaling pathway, etc. The results of KEGG enrichment analysis suggest that vitamin D3 may prevent CRC mainly through the above pathway.

### 3.4 Drug-target-pathway network

**FIGURE 5:**
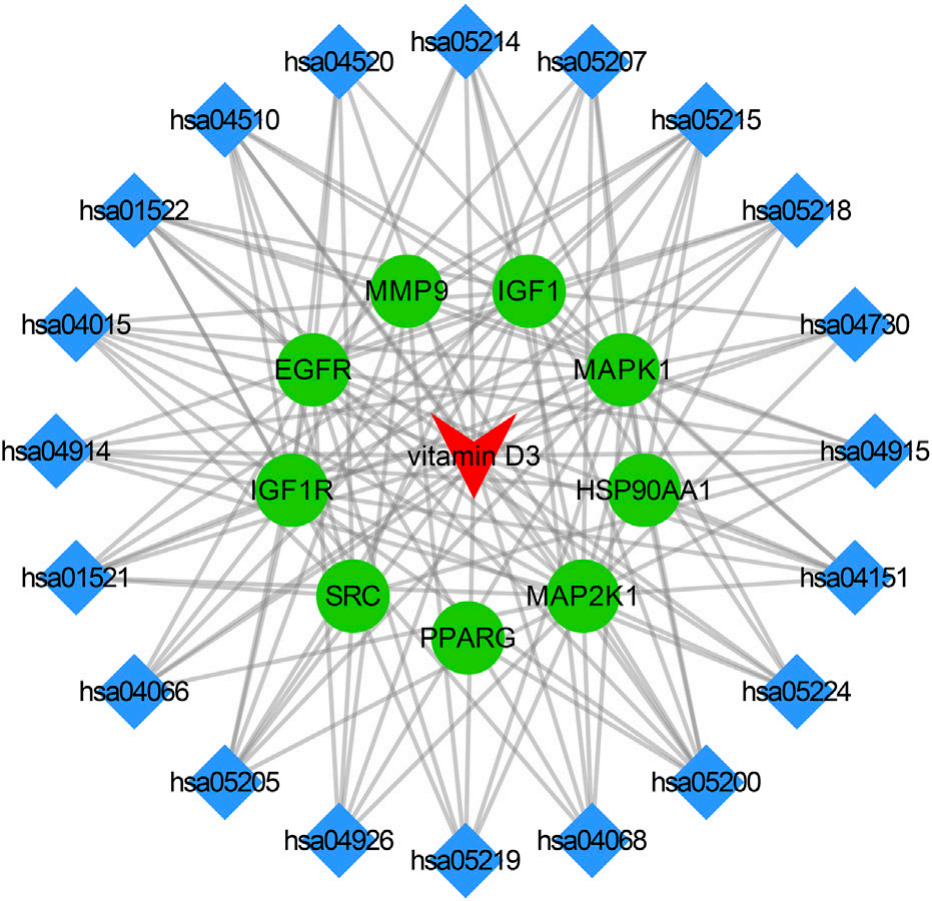
Visualization results of drug-pathway-target relationship network.

The core targets, vitamin D3 and KEGG enrichment pathways were categorized and organized to construct the drug-target-pathway network in Cytoscape 3.9.0 (Figure 5). In Figure 5, the triangle represents vitamin D3, the circle represents the core target, and the diamond represents the pathway in which the core target was enriched.

### 3.5 Results of molecular docking

**FIGURE 6:**
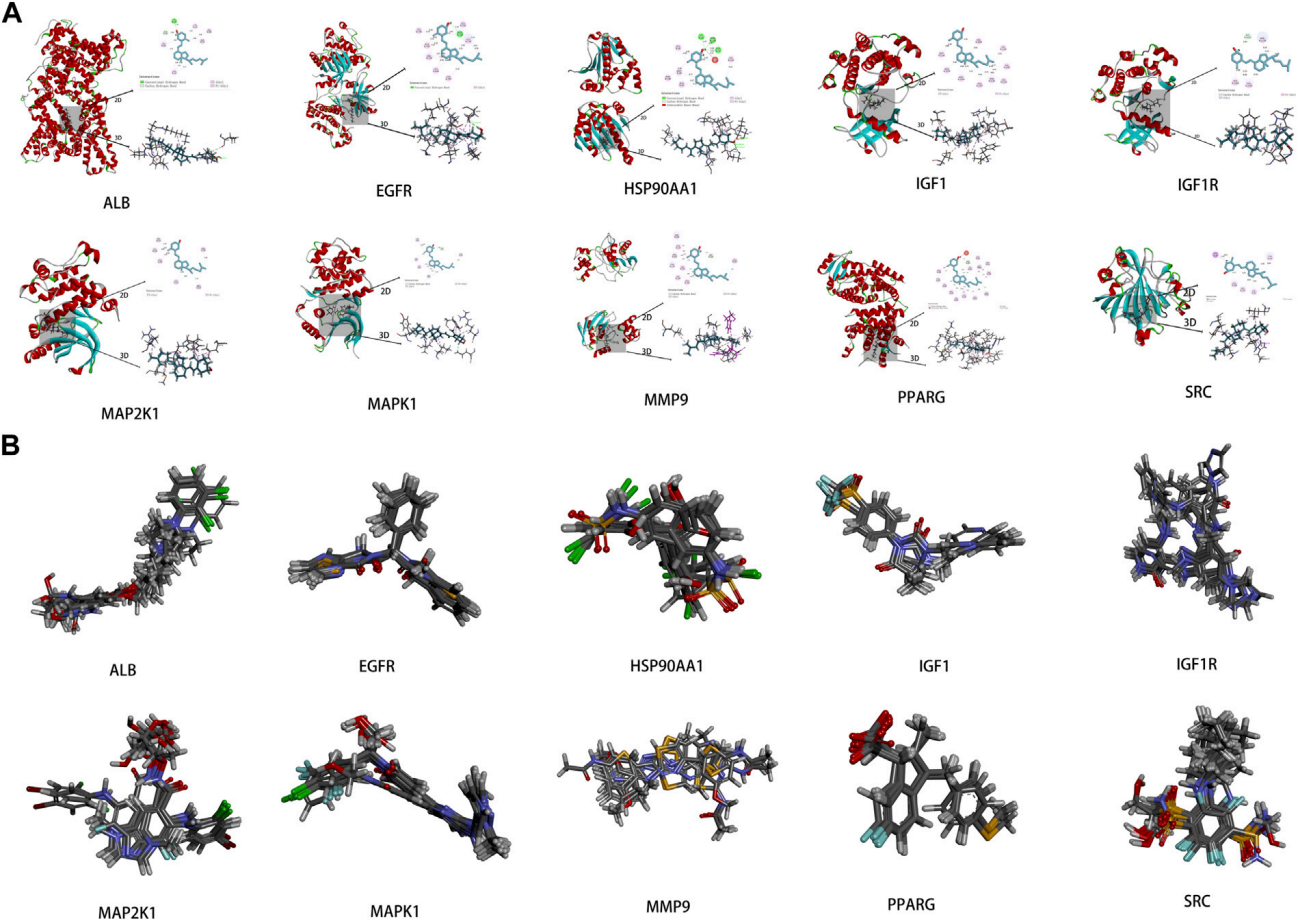
Results of molecular docking **(A)**. Schematic diagram of molecular docking of the core targets with vitamin D3 **(B)**. Visualization results of molecular docking RMSD validation.

To investigate the interaction between the 10 core targets and vitamin D3, molecular docking was used. The relevant information of 10 core target proteins was downloaded from the PDB database (Table 4). First, the 3D structure file of vitaminD3 was uploaded to Discovery Studio 2019; then, its docking potential with ALB, SRC, MMP9, PPARG, HSP90AA1, IGF1, EGFR, MAPK1, MAP2K1 and IGF1R were analyzed. The results of molecular docking scores were obtained. The root-mean-square deviation (RMSD) of the ligand conformation after docking and the ligand conformation of the original crystal structure were calculated. It is generally considered that when the RMSD value is less than 2.0, it proves that the docking method can better reproduce the original ligand-receptor binding mode and the docking parameters are reasonably set (Table 5). All results of RMSD are displayed. The results of core target docking with vitamin D3 are shown, the relationship between vitamin D3 and docking sites was represented in two and three dimensions, respectively (Figure 6A). RMSD validates the molecular docking reliability. In the 2D and 3D structure diagrams, we can observe the type of interaction forces between vitamin D3 and amino acid residues, and the length of the bonds. Validation of the reliability of molecular docking results (Figure 6B). The higher the degree of overlap of different conformations of the same protein, the more reliable the molecular docking results. The RMSD value is generally considered to be less than 2, and molecular docking is more reliable.

**TABLE 4:** Information about core target proteins.

Targets	PDB ID	Method	Resolution (Å)	R-Value free	R-Value work	R-Value observed
ALB	6A7P	X-RAY DIFFRACTION	2.28	0.255	0.207	0.211
SRC	6QL3	X-RAY DIFFRACTION	1.35	0.156	0.116	0.12
MMP9	5UE4	X-RAY DIFFRACTION	1.80	0.224	0.199	0.2
PPARG	4XUH	X-RAY DIFFRACTION	2.22	0.23	0.186	0.189
HSP90AA1	2BZ5	X-RAY DIFFRACTION	1.90	0.254	0.195	0.198
IGF1	3O23	X-RAY DIFFRACTION	2.10	0.278	0.238	——
EGFR	5D41	X-RAY DIFFRACTION	2.31	0.207	0.174	0.175
MAPK1	5K4I	X-RAY DIFFRACTION	1.76	0.232	0.181	0.183
MAP2K1	4U7Z	X-RAY DIFFRACTION	2.81	0.214	0.161	0.163
IGF1R	2OJ9	X-RAY DIFFRACTION	2.00	0.276	0.214	0.217

**TABLE 5:** Results of data related to the docking of vitamin D3 with core target molecules.

Molecular name	Targets	PDB ID	LibDockScore	RMSD(Å)
VitaminD3	ALB	6A7P	159.048	1.32
VitaminD3	SRC	6QL3	120.344	1.29
VitaminD3	MMP9	5UE4	115.312	0.39
VitaminD3	PPARG	4XUH	135.064	0.70
VitaminD3	HSP90AA1	2BZ5	102.392	1.68
VitaminD3	IGF1	3O23	131.303	1.76
VitaminD3	EGFR	5D41	142.962	0.50
VitaminD3	MAPK1	5K4I	135.699	0.41
VitaminD3	MAP2K1	4U7Z	123.954	0.68
VitaminD3	IGF1R	20J9	125.226	1.67

### 3.6 External validation of core targets

#### 3.6.1 The mRNA expression levels of core targets

**FIGURE 7:**
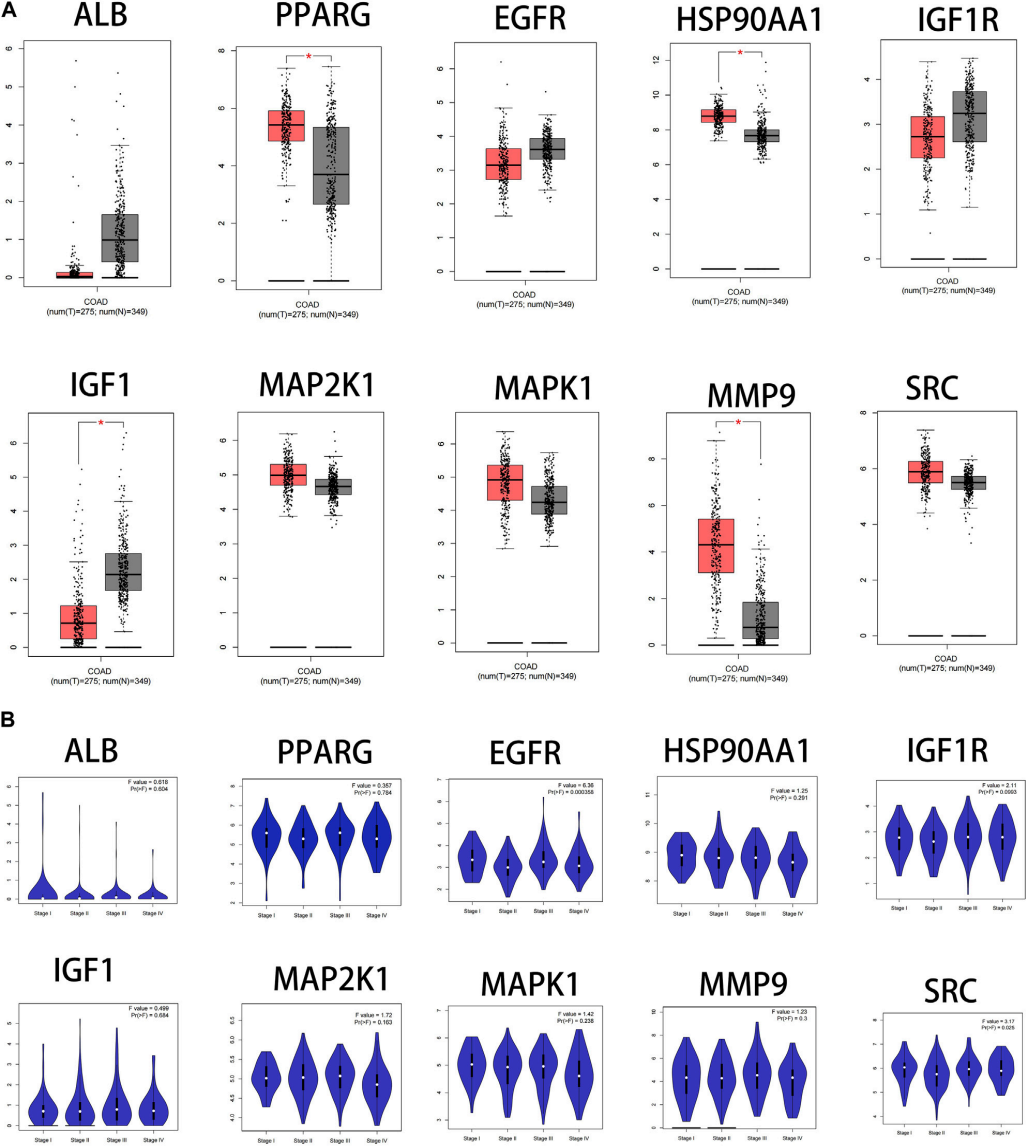
Fact sheet of the core targets on the GEPIA website. **(A)** Boxplot results of mRNA expression levels of core targets. Tumor tissue is shown in red and normal tissue is shown in gray. **(B)** Diagram of the relationship between core target gene expression and CRC cancer stage.

The results showed that the mRNA expression levels of PPARG, HSP90AA1and MMP9 were higher than normal in CRC(*p* < 0.05). The mRNA expression level of IGF1 was lower than normal in CRC (*p* < 0.05) (Figure 7A). The mRNA expression levels of EGFR and SRC correlated with the stages of CRC(*p* < 0.05) (Figure 7B).

#### 3.6.2 The protein expression level of core targets

**FIGURE 8:**
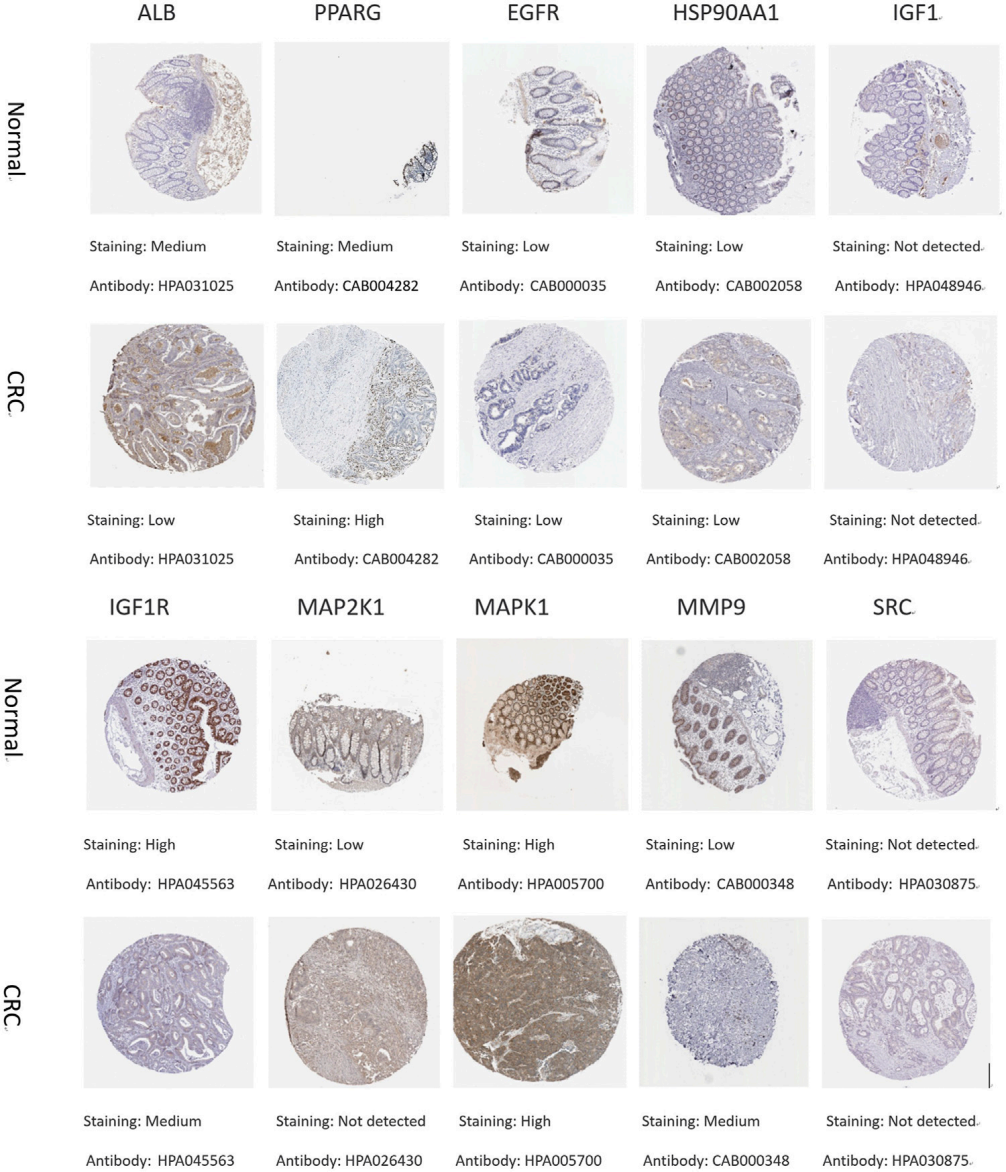
Immunohistochemical pictures of core target proteins in normal and CRC patients from the HPA database.

To understand the expression of core targets in CRC, the relevant immunohistochemical images were analyzed by HPA database. The protein levels of PPARG and MMP9 were found to be high in CRC by comparative analysis. The protein expression of ALB, IGF1R, and MAP2K1 was higher in normal tissues than in CRC. The protein expression of EGFR, HSP90AA1, and MAPK1 was equal in normal and CRC. Immunohistochemical data of IGF1 and SRC were not found in the HPA database (Figure 8).

### 3.7 Genetic alteration of core targets

**FIGURE 9:**
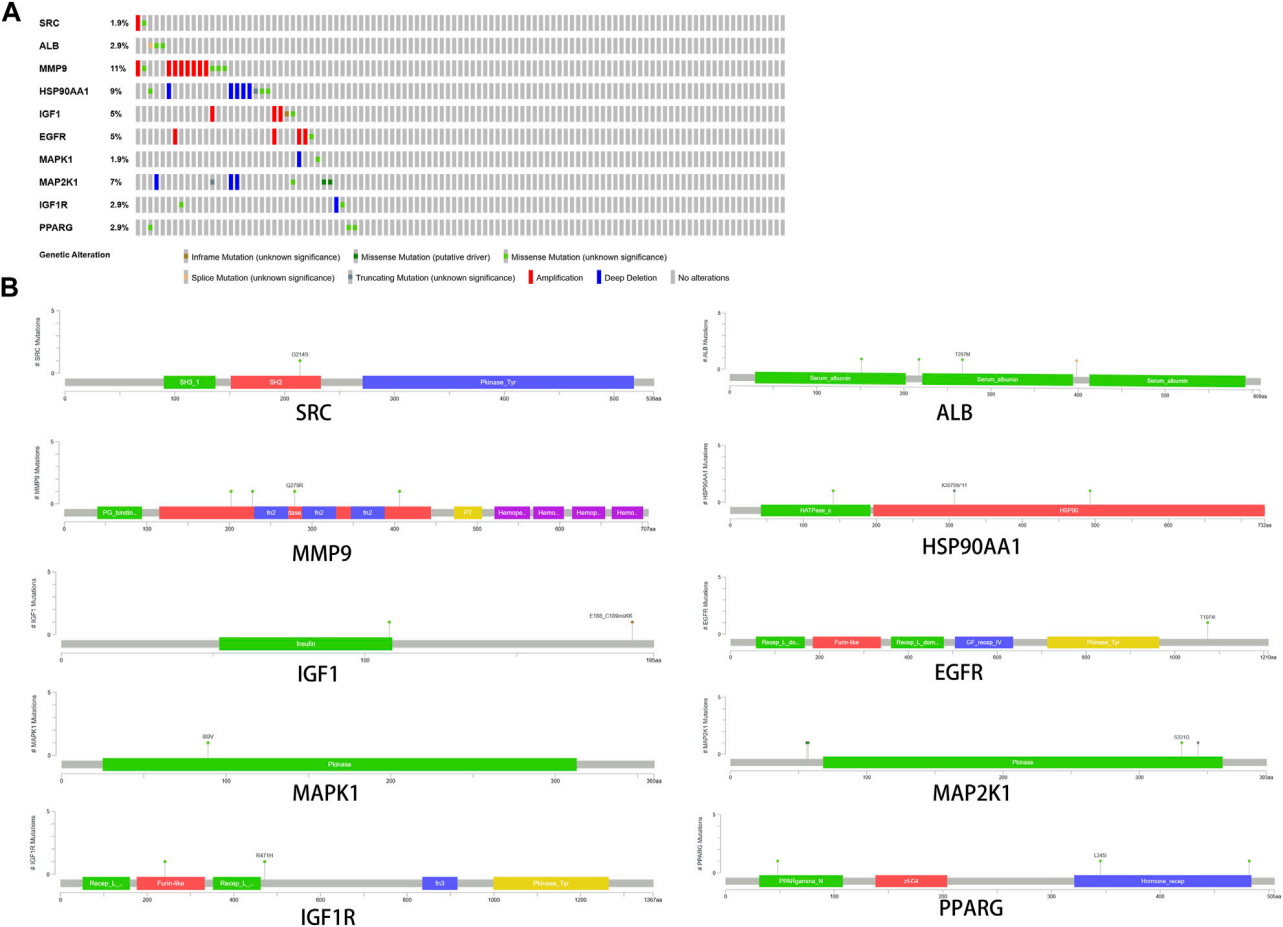
Genetic data on core targets. **(A)** The data suggested that 34 of 105 patients (36%) had a heritable variation on core target genes. **(B)** Information on the site and type of gene mutations on core targets in CRC patients.

Among the samples with mutation and CNA data (105 samples/patient), a total of 36 samples (34%) had mutations in the relevant genes, including 10 core targets (Figure 9A). It also shows the site and type of mutations in the core target genes in CRC patients (Figure 9B). Some details of the mutations in the core target genes are shown (Table 6).

**TABLE 6:** Detailed information about mutations on core target genes.

Gene name	Sample ID	Protein change	Mutation type	Variant type	Start pos	End pos
SRC	11CO018	G214S	Missense	SNP	36024651	36024651
ALB	11CO070	X398_splice	Splice	SNP	74281975	74281975
MMP9	11CO051	A202T	Missense	SNP	44639644	44639644
HSP90AA1	05CO041	T141M	Missense	SNP	102552660	102552660
IGF1	05CO050	Y108N	Missense	SNP	102813367	102813367
EGFR	14CO003	T1074I	Missense	SNP	55270268	55270268
MAPK1	11CO036	I89V	Missense	SNP	22161990	22161990
MAP2K1	11CO019	Q56P	Missense	SNP	66727451	66727451
IGF1R	20CO003	A241T	Missense	SNP	99434634	99434634
PPARG	05CO015	V48M	Missense	SNP	12421262	12421262

### 3.8 The expression of core targets in different cancers

**FIGURE 10:**
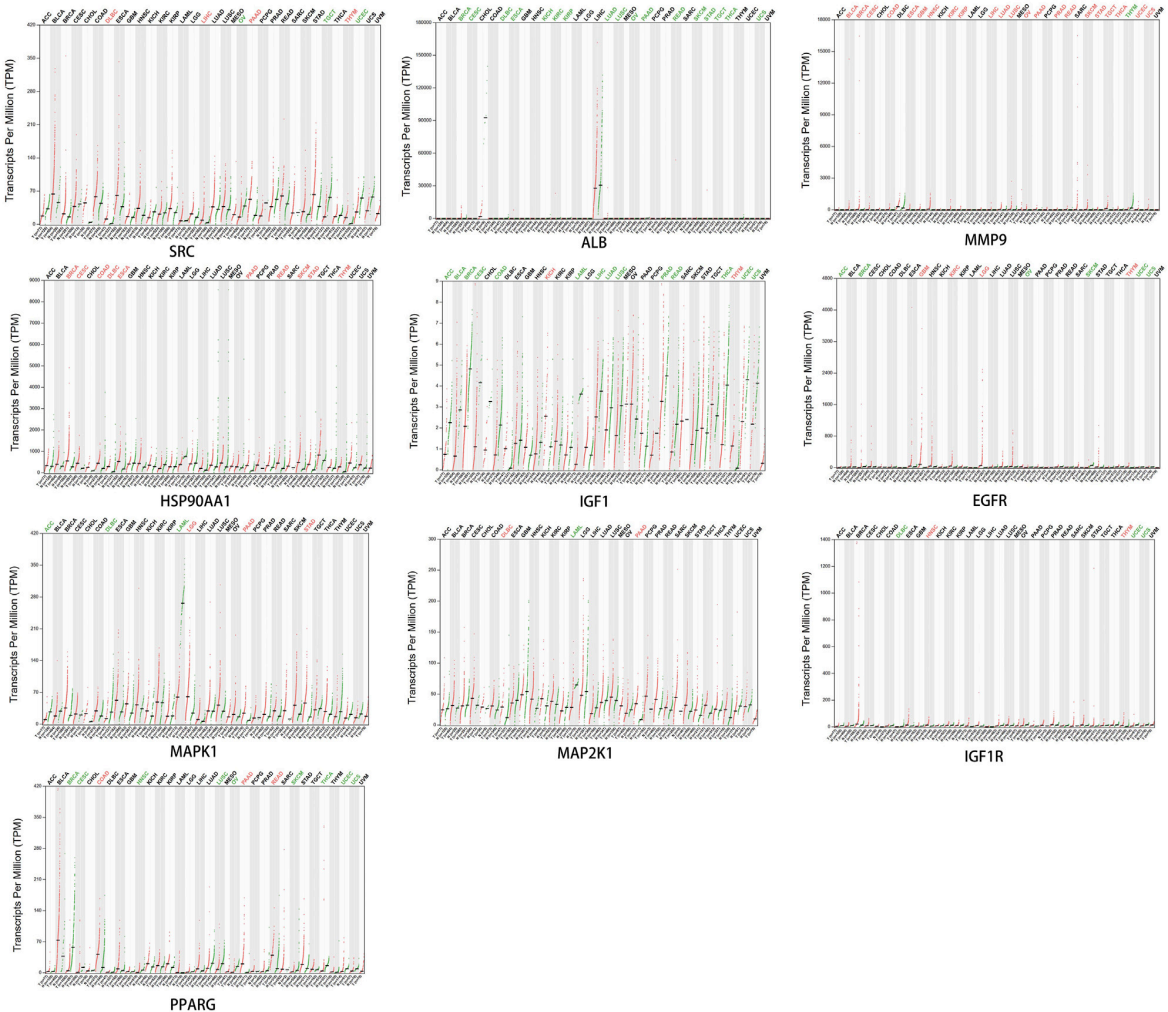
Expression of core target proteins in different cancers.

Dot plots of core target protein distribution were obtained in different cancers in the GEPIA database. The results showed that the expression levels of core target proteins differed in different cancers (Figure 10).

### 3.9 Effect of core targets on overall survival

**FIGURE 11:**
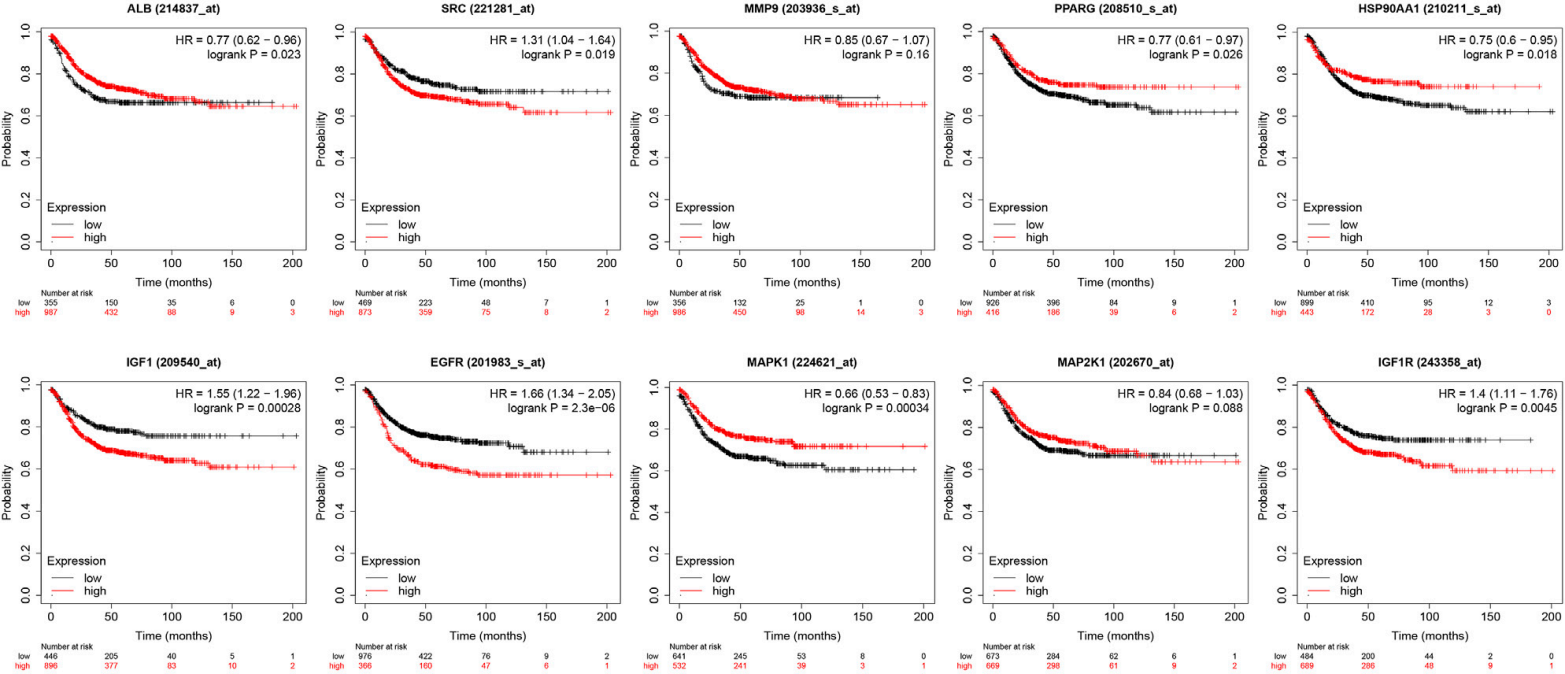
Presentation of results from survival analysis.

The core targets were entered into Kaplan-Meier Plotter for analysis. The effect of expression levels of 10 targets on the overall survival of CRC patients were obtained (Figure 11). The results of survival analysis showed that patients with high expression of ALB,HSP90AA1, PPARG and MAPK1 had a longer survival period than those with low expression, and the difference was statistically significant. Patients with low expression of EGFR,IGF1, IGF1R and SRC in CRC patients had a longer survival time than those with high expression, and the difference was statistically significant. Patients with higher expression of MAP2K1 and MMP9 in CRC had longer survival, but the difference was not statistically significant.

### 3.10 RT-qPCR results

**FIGURE 12:**
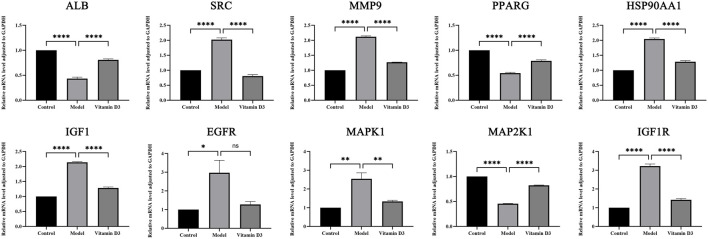
Results of RT-qPCR statistical analysis.

Through the experiment, the following findings were obtained:SRC, IGF1, IGF1R, HSP90AA1, MAPK1 and MMP9 are high expression in CRC tissues and after the intervention of vitamin D3, the expression of these genes was reduced in cancer tissues, and the differences were statistically significant (*p* < 0.05). The expression of EGFR is high in CRC tissues and decreased in CRC tissues after vitamin D3 intervention, but the difference was not statistically significant (*p* > 0.05). ALB, MAP2K1, and PPARG are low expression in CRC tissues, and their expression levels increased after the intervention of vitamin D3, which indicates that the treatment of vitamin D3 is effective and the difference is statistically significant (*p* < 0.05). The results of statistical analysis of RT-qPCR experiments are shown (Figure 12).

## 4 Discussion

The disease burden of CRC has been high and has increased in Asia in recent years (Onyoh et al., 2019). Vitamin D3 is a small molecule compound. More and more epidemiological studies have shown that vitamin D3 has a more prominent role in preventing cancer, especially CRC. The chemopreventive effect of vitamin D3 on CRC has been reported (Lamprecht and Lipkin, 2003). However, the pathogenesis of cancer is complex and the chemical nature of the drug is not fully clear, so the molecular mechanism of the interaction between vitamin D3 and CRC needs to be further investigated.

In this study, we further investigated the molecular mechanism of vitamin D3 in the prevention of CRC through network pharmacology and animal experimental studies. The results of the enrichment analysis suggest that vitamin D3 may play a role in the prevention of CRC through the PI3K-Akt signaling pathway and the HIF signaling pathway. A study showed that vitamin D3 can inhibit cell proliferation, invasion, and metastasis and promote apoptosis through the PI3K-Akt pathway (Songyang et al., 2021). Vitamin D3 can also interfere with the metabolism of cancer cells, inhibit the growth and proliferation of cancer cells, and play an anti-cancer role through the HIF signaling pathway (Gkotinakou et al., 2022).

The mechanisms of action of vitamin D include inhibition of VDR-mediated IGF1, HIF1, NF-κB and PI3K/AKT activities, or activation of MAPK family-related genes, downregulation of cell cycle progression, anti-inflammation, inhibition of angiogenesis, induction of apoptosis, and other anti-tumor effects (Skrajnowska and Bobrowska-Korczak, 2019). Solid tumor cells usually exhibit reduced oxygen levels depending on the rate of cell proliferation, vascular abnormalities, and the distance to the area surrounding the oxygen-containing vessels (Semenza, 2021). The growth and reproduction of cancer cells depend on a moderate supply of oxygen, so a hypoxic microenvironment would significantly affect the biological behavior of cancer cells during their life course. Under hypoxic conditions, the HIF signaling pathway is activated, causing transcription of the corresponding target genes and promoting the development of tumors. Thus, overexpression of HIF-1α can induce increased mortality in many common cancers (Keith et al., 2011). Activation of HIFs induces a series of cascade responses, including alteration of the metabolism of cancer cells and inhibition of the apoptosis of cancer cells. Under the combined effect of these factors, the proliferation, angiogenesis, immune escape, epithelial-mesenchymal transition and the ability of extracellular matrix remodeling of cancer cells are enhanced (Mylonis et al., 2021; Semenza, 2021). The existing evidence suggests that ossifying triol can act on the signaling process of HIF pathway, blocking signal transduction, promoting apoptosis of cancer cells and exerting tumor suppressive effects (Gkotinakou et al., 2022). Mitogen-activated protein kinase (MAPK) is an important member of the serine-threonine kinase family and is the main cell proliferation signaling pathway from the cell surface to the nucleus (Dong et al., 2002).

A growing number of evidences suggest that activation of ERK MAPK pathway is closely associated with the occurrence, development and oncogenic behavior of human CRC (Wang et al., 2004). The antitumor effects of vitamin D in CRC are achieved through various pathways such as promoting cell cycle arrest in the G1 phase, reducing the synthesis of vascular endothelial growth factor (VEGF), and decreasing the ability of cell migration and angiogenesis. Meanwhile, IGF1 and IGF1R can induce cell proliferation, promote the transcription and expression of vascular endothelial growth factor genes, promote angiogenesis, provide more nutrients for tumor growth, and promote the further development of CRC to advanced stages. The effect of vitamin D on the G1/S cell cycle transition is opposite to that of IGF-1 and also has antagonistic effects on angiogenesis and the tumor microenvironment. Therefore, this evidence may provide new ideas on the mechanism of the prevention of vitamin D in CRC (Ciulei et al., 2020). The results of RT-qPCR from animal experiments in this study also indicated that vitamin D has a therapeutic and preventive effect on CRC.

These results suggest that vitamin D may be an effective chemical for the prevention of CRC. In summary, ALB, SRC, MMP9, PPARG, HSP90AA1, IGF1, EGFR, MAPK1, MAP2K1 and IGF1R play important roles in the pathogenesis of CRC. Therefore, based on the network pharmacological analysis, it is expected to discover more novel compounds that can prevent cancer or play an anti-cancer role. This study provides additional theoretical basis for the prevention of CRC with vitamin D3.

In this study, vitamin D was found to be a multi-target medicine for the prevention of CRC. We predict that the main mechanism of vitamin D for the prevention of CRC is the regulation of tumor cell proliferation, apoptosis, migration and angiogenesis through signaling pathways such as HIF-1, FoxO and PI3K-Akt, thus playing a role in the prevention of CRC.

## 5 Conclusion

Based on network pharmacological analysis, this study predicted 10 potential core targets of vitamin D3 for the prevention of CRC, suggesting that vitamin D3 is a single-component, multi-target, multi-pathway small molecule compound. Network pharmacological analysis suggests that vitamin D3 may play a role in CRC chemoprevention by regulating different targets, such as MAPK, IGF1 and IGF1R. Graphene oxide analysis of the core targets ALB, SRC, MMP9, PPARG, HSP90AA1, IGF1, EGFR, MAPK1, MAP2K1 and IGF1R showed that vitamin D3 exerts pharmacological effects by affecting different biological processes, such as endosomal transport and positive regulation of protein kinase B signaling. The analysis of KEGG pathway in this study suggests that vitamin D3 may exert pharmacological effects by simultaneously regulating different CRC-related signaling pathways, such as FoxO signaling pathway, HIF-1 signaling pathway, PI3K-Akt signaling pathway, etc. These results support that vitamin D3 may mediate the inhibition of HIF-1 through inhibition of the upstream PI3K-AKT pathway.

In summary, vitamin D3 may be a new safe, effective, multi-targeted medicine for the prevention of CRC. This study concludes from a network pharmacological analysis that vitamin D3 may play a preventive role in CRC by regulating cell metabolism, proliferation and apoptosis through multiple targets, pathways and biological processes. However, the clinical efficacy of vitamin D3 in the prevention of CRC and its mechanism of action need to be further validated.

## Data Availability

The original contributions presented in the study are included in the article/Supplementary Material, further inquiries can be directed to the corresponding author.
